# Relational conflicts during COVID-19: Impact of loss and reduction of employment due to prevention measures and the influence of sex and stress (in the iCARE study)

**DOI:** 10.1177/13591053241260672

**Published:** 2024-08-15

**Authors:** Noémie Tremblay, Camille Leger, Frédérique Deslauriers, Lydia Hébert-Auger, Vincent Gosselin-Boucher, Simon L. Bacon, Maximilien Vakambi Dialufuma, Kim L. Lavoie, for the iCARE Study Team

**Affiliations:** 1 Departement of Psychology, Université du Québec à Montréal, Canada; 2 Montreal Behavioural Medicine Centre, CIUSSS du Nord-de-l’Ile-de-Montreal, Canada; 3 School of Kinesiology, University of British Columbia, Canada; 4 Department of Health, Kinesiology and Applied Physiology, Concordia University, Canada

**Keywords:** COVID-19-related stress, domestic violence, financial problems, impacts of the pandemic, sex inequalities

## Abstract

This study explored the association between pandemic-related loss/reduction of employment, sex, COVID-19-related stress and relational conflicts. A sample of 5103 Canadians from the iCARE study were recruited through an online polling firm between October 29, 2020, and March 23, 2021. Logistic regressions revealed that participants with loss/reduction of employment were 3.6 times more likely to report increased relational conflicts compared to those with stable employment (OR = 3.60; 95% CIs = 3.03–4.26). There was a significant interaction between employment status and sex (*x*^2^ = 10.16; *p* < 0.005), where loss/reduction of employment was associated with more relational conflicts in males compared to females. There was a main effect of COVID-19-related stress levels on relational conflicts (increased stress vs no stress : OR = 9.54; 95% CIs = 6.70–13.60), but no interaction with loss/reduction of employment (*x*^2^ = 0.46, *p* = 0.50).

## Background

The coronavirus disease 2019 (COVID-19) outbreak was declared a global pandemic on March 11, 2020, by the World Health Organization (Généreux et al., 2020). As of February 2024, 7 million deaths and >774 million cases in 215 countries have been reported (World Health Organization, 2024). To reduce the spread of the virus, governments and health authorities implemented several preventive measures, such as social distancing, mandatory self-isolation and quarantine, that have been associated with several psychosocial consequences, which were not the priority of these measures during the pandemic crisis (Alon et al., 2020; Bahire et al., 2022; Balzarini et al., 2023; Gresham et al., 2021; Langhinrichsen-Rohling et al., 2022; Lee et al., 2021; Shahabi et al., 2023; UN Women, 2021; Weber et al., 2021; Xue et al., 2021; Béland et al., 2022; Blix, Birkeland & Thoresen, 2021). Due to restrictions in social mobility, people had to spend more time at home with their families, in addition to experiencing long periods of social deprivation, which may have increased the risk of familial conflict (Balzarini et al., 2023). Supporting this, several studies have reported lower relationship satisfaction within couples and an increase in relational conflicts during the pandemic, primarily due to stress, social isolation, job losses and financial problems (Bahire et al., 2022; Balzarini et al., 2023; Langhinrichsen-Rohling et al., 2022).

The pandemic also led to a high number of job losses among workers, in addition to a major economic slowdown in Canada (Alon et al., 2020; Deady et al., 2020; Béland et al., 2022). The Canadian unemployment rate rose to 13% in April 2020 from 7.8% in March 2020 to 5.6% in February 2020 (Béland et al., 2020, 2022). An American study demonstrated an increased risk of relational conflict among people who experienced job loss during the COVID-19 pandemic because of its association with feelings of stress and anger (Langhinrichsen-Rohling et al., 2022). The negative emotions associated with job losses can result in depressive symptoms in both males and females and ultimately increase marital dissatisfaction and disputes (Howe et al., 2004). The results of Langhinrichsen-Rohling et al. (2022) study were partially attributed to the loss of social support and health insurance resulting from job losses (Langhinrichsen-Rohling et al., 2022). Having health insurance seemed to correlate with increased family stability, likely due to reduced stress levels and the sense of security it provides (Langhinrichsen-Rohling et al., 2022). Given the unique healthcare system and the labour market in Canada, characterized by features such as universal access to healthcare, it would be crucial to look at the effects of job loss and financial strain on relational conflicts in this specific context. Furthermore, job losses can amplify financial strain, increase negative emotional states and lower life satisfaction, impact relationship quality, as well as elevate the risk of conflicts between partners (Conger et al., 1999; Ervasti and Venetoklis, 2010; Randall and Bodenmann, 2009; Weber et al., 2021; Williamson et al., 2013).

The literature also illustrates that females were disproportionately affected by COVID-19 and its preventive measures (Alon et al., 2020; Lee et al., 2021; Reichelt et al., 2021; UN Women, 2021; Xue et al., 2021). Indeed, COVID-19 significantly affected service sectors such as restaurants, retail, hospitality and tourism, as well as the healthcare sector, which employs a higher proportion of female workers (Alon et al., 2020; UN Women, 2021). According to Statistics Canada (2022b), females comprise 54.9% of the workforce in the sales and service sector, while 79.4% healthcare sector are female. Also, with the closure of schools and daycares during lockdown periods, the burden of childcare fell more heavily on mothers, which led to greater mental health issues such as psychological distress (Alon et al., 2020; Lee et al., 2021; Reichelt et al., 2021; Williamson et al., 2013; Xue et al., 2021). Research also showed that the economic consequences of school and daycare closures were particularly devastating for females in lower financial positions (UN Women, 2021). Some females had to reduce their working hours or temporarily quit their jobs to care for their children and elderly parents, disproportionately impacting those with lower socioeconomic status who are already struggling with income instability (UN Women, 2021; Collins et al., 2021; Power, 2020). Additionally, Henke and Hsu (2022) demonstrated that the COVID-19 pandemic significantly increased reports of domestic violence in several countries, due to mandatory stay-at-home policies and increased unemployment. Exploring the opportunity and temptation for crime, the Exposure Reduction Theory posits that increased exposure to opportunities for crime leads to a higher frequency of criminal activity (Henke and Hsu, 2022). The pandemic-induced shutdowns and the imperative to stay home for infection control created an exceptional circumstance where both abusers and victims found themselves confined together throughout the day, thereby escalating the likelihood of abuse (Henke and Hsu, 2022). Henke and Hsu (2022) showed that the exposure effect was strongest when people began to stay at home starting in March 2020 because of the ‘shock’ of the lockdown, with the effect beginning to fade afterwards. According to UN Women (2020), since the outbreak of COVID-19, domestic violence and demands for emergency shelters have intensified in Canada, Germany, Spain, the United Kingdom and the United States. In France, reports of domestic violence have increased by 30% since the lockdown on March 17th (UN Women, 2020). The rise of conflicts can also be observed with unemployment, as job loss can increase family stress and potentially lead to more violence (Henke and Hsu, 2022). Aligned with this, the Household Bargaining Theory posits that job losses among females in heterosexual relationships could contribute to an uptick in domestic violence due to a shift in economic bargaining power favouring males (Anderberg et al., 2016; Béland et al., 2020; Bowlus and Seitz, 2006; Henke and Hsu, 2022).

Numerous studies have demonstrated the influence of COVID-19 preventive measures on relational conflicts and have identified certain risk factors, including job loss, financial difficulties, stress and sex disparities (Alon et al., 2020; Bahire et al., 2022; Balzarini et al., 2023; Gresham et al., 2021; Langhinrichsen-Rohling et al., 2022; Lee et al., 2021; Shahabi et al., 2023; UN Women, 2021; Xue et al., 2021). While most studies have concentrated on conflicts and disputes within couples, our research delves into relational conflicts involving domestic violence (physical and verbal altercations) among family members residing under the same roof. Understanding which specific factors contributed to the increase in conflicts during the COVID-19 pandemic is essential for making recommendations to governments and health authorities regarding strategies and intervention programmes in the event preventive measures are reinstated due to the emergence of new COVID-19 variants or future pandemics. Therefore, the objectives of this study were to determine if loss or reduction of employment was associated with an increase in relational conflicts (physical and verbal fights with people in the household) and the extent to which this relationship was influenced by participant’s sex and by COVID-19-related stress levels in a sample of Canadian adults.

## Methods

### Study design

This study represents a sub-analysis of the International COVID-19 Awareness and Responses Evaluation (iCARE) Study lead by members of the Montreal Behavioural Medicine Centre (MBMC: https://mbmc-cmcm.ca/, accessed on 10 November 2022). This international, cross-sectional, multi-wave observational study aims to examine public awareness, attitudes and responses to COVID-19 public health policies through a series of online surveys. The study protocol and detailed methods have been published elsewhere (Bacon et al., 2021; Lavoie et al., 2022). The iCARE study was approved by the Research Ethics Board of the Centre Intégré Universitaire de Santé et de Services Sociaux du Nord-de-l’île-de-Montréal (CIUSSS-NIM), REB#: 2020-2099/25-03-2020.

### Study participants and recruitment

For this sub-study, we analysed three rounds of the Canadian representative sample, which were collected between October 29, 2020, and March 23, 2021 (surveys 3–5, *N* = 9011). These data, covering the second wave of the pandemic and the early stages of vaccination, were selected to adequately capture periods of employment changes due to job losses and income reduction. This 6-month timeline allows for the assessment of the medium-term effects of job/income losses on the Canadian population.

To gather reliable data on the Canadian population, we collected data from cross-sectional samples weighted by age, sex and province; samples were recruited from an online panel consisting of Canadians aged 18 years and older. The polling firm (Léger Opinion©, one of the largest in Canada) recruits participants through their closed proprietary online panel (LégerWeb.com) which includes over 400,000 Canadians, the majority of which (61%) were recruited within the past 10 years. Two-thirds of the panel were recruited randomly by telephone, with the remainder recruited via publicity and social media. All respondents were invited to voluntarily complete the survey via email and provided with a unique link to ensure they could not complete the survey more than once. Using data from Statistics Canada, a weighting variable was created which was weighted within each province according to the sex and age of the respondents to make their profiles representative of the current population within each Canadian province (excluding the three territories). The weight of each province was further adjusted to represent their actual weight within the 10 Canadian provinces.

### ICARE survey

At the outset of the analysis across the three surveys, 9011 participants were recruited. Participants were included (*n* = 5103) based on their reported employment status before the COVID-19 pandemic, as determined by the question: ‘Prior to the COVID-19 pandemic, how would you describe your employment status?’ Those identified as ‘part-time employees (*n* = 998)’, ‘full-time employees (*n* = 3649)’ and ‘self-employed (*n* = 530)’ were included, while individuals categorized as ‘retired, homemaker (*n* = 2426)’, ‘receiving social assistance or on disability pay (*n* = 451)’, ‘student (*n* = 787)’, ‘unemployed (*n* = 480)’ and ‘I don’t know/I prefer not to answer (*n* = 87)’ were excluded (*n* = 3908). Please note that the differences in total frequencies compared to those categorized by employment status are due to the weighted variable implemented to ensure sample representativeness.

The survey includes approximately 75 questions and takes between 15 and 20 minutes to complete. The survey included questions about sociodemographics, physical and mental health, prior COVID-19 infection, general health behaviours, perceptions and attitudes about local COVID-19 prevention policies, concerns about the virus and its impacts, and vaccine attitudes, intentions, motivations and behaviours. A detailed description and copy of all surveys can be found at the following weblink (https://osf.io/nswcm/). For the present report, we analysed the following variables: sociodemographics; and self-reported impacts (relational conflicts, loss or reduction of employment and COVID-19-related stress levels). For more information on the list of measures available in the iCARE study, as well our data dictionaries, see https://mbmc-cmcm.ca/mbmc/covid19/apl/.

The ‘Impacts’ module of the iCARE survey was used to assess changes in employment status, relational conflicts and COVID-19-related stress levels. The participants were asked the following question: ‘Please indicate the impact that COVID-19 has had on the following aspects of your life in the past month’ (Possible responses: ‘strongly’, ‘somewhat’, ‘very little’, ‘not at all’, ‘I don’t know/I prefer not to answer’, ‘does not apply’). Relational conflicts were assessed by the question: ‘I had more physical and verbal altercations with family members I live with’. Loss or reduction of employment was assessed by the questions: ‘I lost my job or had to close my business’ and ‘I saw a reduction in working hours/lost income’. The COVID-19-related stress level was assessed by the questions: ‘I have felt nervous, anxious or worried’, ‘I have felt sad, depressed or hopeless’ and ‘I have felt irritable, frustrated or angry’. For all impacts assessed, participants who responded “strongly” or “somewhat” to any statements were classified as having experienced loss or reduction of employment, relational conflicts and COVID-19-related stress. Responses of ‘I don’t know/I prefer not to answer’ were treated as missing values and not included in the analyses.

### Data analysis

The weighted sociodemographic characteristics of the overall sample (province, region, age, sex, ethnicity, average income, education, pre-pandemic employment status, current employment status, professional sector, COVID-19-related stress level, any anxiety/depressive disorders) were summarized using descriptive analyses. Before conducting our main analyses, we performed correlation and multicollinearity analyses to ensure that variables were not strongly correlated with each other. Subsequently, logistic regression analyses were used to assess the association between loss or reduction of employment (independent variable) and relational conflicts (dependent variable) in the main effect model. Following this, a second model was employed, retaining the same variables as above and introducing an interaction term between sex and loss or reduction of employment. Given the significance of the interaction, disaggregated analyses were conducted for males and then for females to explore potential differences or patterns that may exist within each subgroup, providing a more nuanced understanding of the data and its implications. An additional model explored the associations between COVID-19-related stress levels and relational conflicts. Bonferroni corrections were applied to mitigate the risk of having false positive results (type I errors), and the results remained unchanged for both models (sex and COVID-19-related stress levels). Models were adjusted for covariates including: age; survey wave; ethnicity (white vs other); the presence of anxiety and/or depressive disorders; sex (for all models except sex specific analyses); number of children in the household; and the weighting variable. All statistical tests were two-tailed, and a *p*-value < 0.05 was considered statistically significant. Statistical analyses were conducted using SAS, version 9.4 (SAS Institute Inc., Cary, North Carolina, United States).

## Results

### Full sample description

A summary of participant characteristics for surveys 3–5 is presented in Table 1. The sample consisted of 51.3% males, 56.3% were aged between 26 and 50 years old and most participants (65.9%) had a high school diploma or lower education level. Over half of the participants had an average income of over 60,000 CAD (60.5%), and 66.4% of the sample reported not living with children. Regarding employment status, most of the sample (88.1%) were employed when they completed the survey. In terms of mental health, a small portion of the sample reported having a diagnosis for a depressive disorder (16.1%) or an anxiety disorder (21.8%). Across all waves, an average of 70.1% of the participants reported an increase in stress during COVID-19. There was a trend for the proportion of people who reported a high level of stress to decrease from survey 3 to 5 (survey 3: 73%, survey 4: 71.1%, survey 5: 66.2%) but this difference was not statistically significant. 43.5% of participants reported a loss or reduction in employment. Among them, 45.2% of women reported experiencing loss or reduction of employment, compared to 42.0% of men. Moreover, 22.7% of participants reported experiencing relational conflicts, including 21.7% of men and 23.8% of women. A comparison of all data across the three surveys was conducted, and no statistically significant differences were found. Therefore, a single table was used to summarize the descriptive characteristics of surveys 3–5 (the demographics by each survey are available in the Supplemental material, S1).

**Table 1.** Sample’s weighted descriptive characteristics (Surveys 3–5).

Sociodemographic characteristics	General sample (*N* = 4271)	Participants with loss/reduction of employment (*n* = 1858)	Participants without loss/reduction of employment (*n* = 2413)
Variables	% (*n*)		
Sex			
Male	51.3 (2611)	51 (948)	54.2 (1308)
Female	48.7 (2482)	48.9 (909)	45.8 (1104)
Missing values	11	8	1
Age			
51 years old and more	33.9 (1707)	28.8 (530)	34.3 (820)
26–50 years old	56.3 (2835)	56.8 (1047)	59.1 (1413)
Less than 25 years old	9.9 (497)	14.4 (266)	6.6 (158)
Missing values	64	22	24
Education level			
High school diploma or less	65.9 (3311)	66.7 (1217)	63.5 (1523)
College or more	34.1 (1716)	33.3 (608)	36.5 (876)
Missing values	77	42	15
Region (province)			
Ontario	37.4 (1906)	40.7 (760)	34.4 (831)
Quebec	24.6 (1257)	17.5 (327)	29.4 (711)
British Columbia	13.7 (696)	16 (298)	12.6 (303)
Alberta	11.6 (593)	14.4 (268)	10.4 (250)
Saskatchewan	3.2 (161)	2.8 (53)	2.9 (71)
Manitoba	2.9 (149)	2.9 (54)	2.9 (71)
Nova Scotia	2.9 (148)	3.2 (59)	2.7 (65)
New Brunswick	2.2 (114)	1.5 (28)	2.7 (65)
Newfoundland	1.3 (65)	0.9 (17)	1.6 (38)
Prince Edward Island	0.3 (14)	0.1 (2)	0.3 (9)
Region type			
Suburban or suburb	36.1 (35)	46.8 (23)	22.8 (6)
Urban or city	36.1 (35)	33 (16)	45.4 (12)
Rural or countryside	27.9 (27)	20.2 (10)	31.8 (8)
Missing values	5006	1816	2388
Income			
60K/year or more	60.5 (2800)	50.8 (851)	72 (1604)
Less than 60K/year	39.5 (1826)	49.2 (823)	28 (625)
Missing values	1477	192	185
How many children (under 18 years old) live with you at home?			
No children	66.4 (3312)	66.1 (1199)	64.3 (1536)
At least one child	33.6 (1779)	33.9 (614)	35.7 (854)
Missing values	113	53	24
Current employment status			
Employed	88.1 (4377)	77.7 (1395)	97.9 (2328)
Unemployed	11.9 (589)	22.3 (400)	2.1 (50)
Missing values	138	71	35
Main pre-pandemic employment sector			
Professional	23.6 (1034)	20.9 (307)	25.5 (578)
Service and sales worker	17.2 (752)	23.4 (344)	12.9 (292)
Manager	13.6 (595)	14.5 (214)	13.7 (310)
Technician or associate professional	8.8 (383)	6.7 (99)	9.1 (205)
Craft and related trades	4.9 (216)	6.5 (96)	4 (91)
Plant machine operators and assemblers	4.9 (213)	4 (59)	6 (136)
Elementary occupations	4.5 (196)	5.1 (75)	4 (91)
Agriculture, forestry and fishing worker	2 (86)	1.4 (20)	2.5 (57)
Public service worker	1 (45)	0.0 (0)	1.5 (35)
Armed forces occupations	0.8 (34)	0.5 (7)	1.1 (26)
Other	1.4 (62)	2.1 (31)	0.8 (18)
Missing values	721	395	148
Presence of any depressive disorder			
No	83.9 (4151)	79.1 (1427)	88.2 (2104)
Yes	16.1 (794)	20.9 (377)	11.8 (282)
Missing values	158	62	28
Presence of any anxiety disorder			
No	78.2 (3865)	70.8 (1282)	84.5 (2011)
Yes	21.8 (1080)	29.2 (529)	15.5 (369)
Missing values	158	56	33
COVID-19-related stress level			
Increase	70.1 (3503)	86.6 (1605)	57.1 (1375)
No increase	29.9 (1495)	13.4 (248)	42.8 (1031)
Missing values	106	12	8
Loss or reduction of employment			
No	56.5 (2413)		100 (2414)
Yes	43.5 (1858)	100 (1866)	
Missing values	833		
Relational conflicts			
No	77.3 (3518)	62.5 (1060)	87.2 (1976)
Yes	22.7 (1031)	37.5 (636)	12.8 (291)
Missing values	555	170	147

The correlation and multicollinearity analysis findings are detailed in the Supplemental material (S2 and S3). Multicollinearity diagnostics indicated that all variables exhibited variance inflation factors (VIFs) below 10, suggesting the absence of substantial inter-variable correlations.

### Association between loss or reduction of employment and relational conflicts

A summary of the associations between loss or reduction of employment and relational conflicts (and the covariates) are presented in Table 2. Logistic regression analyses revealed that participants reporting loss or reduction of employment were 3.6 times more likely to report an increase in relational conflicts compared to those who did not report loss or reduction of employment (OR = 3.60; 95% CIs = 3.03–4.26). The adequacy of the model was tested with the Hosmer-Lemeshow (*p* > 0.05) and the global model testing (*p* < 0.001).

**Table 2.** Association between loss or reduction of employment and relational conflicts (and covariates)*.

Variables	OR (95% CI)	DF	Wald Chi-Square	*p*-value
**Loss or reduction of employment** (reference: no loss or reduction of employment)	3.60 (3.03–4.26)	1	215.91	<**0.001**
**Sex** (reference: man)	1.02 (0.86–1.20)	1	0.04	0.834
**Age** (reference: 51 years old and more)			31.90	
26–50 years old	1.65 (1.33–2.03)	2		<**0.001**
Less than25 years old	2.16 (1.61–2.90)	2		<**0.001**
**Ethnicity** (reference: white)	1.57 (1.29–1.90)	1	20.60	<**0.001**
**Wave number** (reference: wave 5)			0.35	
3	1.05 (0.85–1.29)	2		0.661
4	1.06 (0.87–1.30)	2		0.572
**Depressive disorder** (reference: no depressive disorder)	1.73 (1.35–2.23)	1	18.28	<**0.001**
**Anxiety disorder** (reference: no anxiety disorder)	1.40 (1.11–1.76)	1	7.94	<**0.005**
**Living with children (under 18** **years old)** (reference: living without children)	1.36 (1.14–1.63)	1	11.62	<**0.001**

OR: odds ratio, CI: confidence interval, DF: degree of freedom.

* Adjusted for age, survey wave, ethnicity (white vs other), the presence of anxiety/depressive disorders, living with children (under 18 years old) and the weighted variable.

### Association between loss or reduction of employment and relational conflicts influenced by sex

Logistic regression analyses revealed a significant interaction between sex and loss or reduction of employment on relational conflicts (*x*^2^ = 10.16, *p* < 0.005, Supplemental material S4). The adequacy of the model was tested with the Hosmer-Lemeshow (*p* > 0.05) and the global model testing (*p* < 0.001). In a model that only included males (Table 3), it was found that males reporting loss or reduction of employment were 5.2 times more likely to report relational conflicts compared to males without such loss or reduction of employment (OR = 5.22; 95% CIs = 4.09–6.67). A model that only included females (Table 4) revealed that females experiencing loss or reduction of employment were nearly 2.4 times more likely to report relational conflicts compared to females without such loss or reduction of employment (OR = 2.38; 95% CIs = 1.87–3.03). Loss or reduction of employment significantly impacted males more than females.

**Table 3.** Association between loss or reduction of employment and relational conflicts (and covariates) in males.

Variables	OR (95% CI)	DF	Wald Chi-Square	*p*-value
**Loss or reduction of employment** (ref: Males without loss/reduction of employment)	5.22 (4.09–6.67)	1	176.05	<**0.001**
**Age** (reference: 51 years old and more)			12.74	
26–50 years old	1.66 (1.25–2.22)	2		<**0.005**
Less than 25 years old	1.72 (1.10–2.73)	2		<**0.005**
**Ethnicity** (reference: white)	1.46 (1.12–1.92)	1	7.63	0.06
**Wave number (**reference: wave 5)			2.53	
3	1.11 (0.84–1.48)	2		0.28
4	0.88 (0.66–1.18)	2		0.28
**Depressive disorder** (reference: no depressive disorder)	1.75 (1.20–2.57)	1	8.07	<**0.005**
**Anxiety disorder** (reference: no anxiety disorder)	1.48 (1.03–2.14)	1	4.47	0.03
**Living with children (under 18** **years old)** (reference: living without children)	1.35 (1.05–1.73)	1	5.52	0.02

OR: odds ratio, CI: confidence interval, DF: degree of freedom.

* Adjusted for age, survey wave, ethnicity (white vs other), the presence of anxiety/depressive disorders, living with children (under 18 years old) and the weighted variable.

**Table 4.** Association between loss or reduction of employment and relational conflicts (and covariates) in females.

Variables	OR (95% CI)	DF	Wald Chi-Square	*p*-value
**Loss or reduction of employment** (ref: Females without loss/reduction of employment)	2.38 (1.87–3.03)	1	48.82	<**0.001**
**Age** (reference: 51 years old and more)			24.68	
26–50 years old	1.66 (1.21–2.28)	2		<**0.001**
Less than 25 years old	2.71 (1.83–4.02)	2		<**0.001**
**Ethnicity** (reference: white)	1.76 (1.33–2.33)	1	15.32	<**0.001**
**Wave number** (reference: wave 5)			4.58	
3	0.97 (0.72–1.31)	2		0.10
4	1.29 (0.97–1.71)	2		0.10
**Depressive disorder** (reference: no depressive disorder)	1.75 (1.26–2.45)	1	10.95	<**0.005**
**Anxiety disorder** (reference: no anxiety disorder)	1.33 (1.00–1.80)	1	3.54	0.06
**Living with children (under 18** **years old)** (reference: living without children)	1.36 (1.05–1.77)	1	5.52	0.02

OR: odds ratio, CI: confidence interval, DF: degree of freedom.

* Adjusted for age, survey wave, ethnicity (white vs other), the presence of anxiety/depressive disorders, living with children (under 18 years old) and the weighted variable.

### Association between loss or reduction of employment and relational conflicts influenced by COVID-19-related stress levels

Logistic regression analyses revealed a significant main effect of COVID-19-related stress levels on relational conflict, such that those who reported higher level of stress were nearly 10 times more likely to report relational conflict (OR = 9.54; 95% CIs = 6.70–13.60). However, there was no interaction between COVID-19-related stress levels and loss or reduction of employment on relational conflicts (*x*^2^ = 0.46, *p* = 0.50). The details about these results can be found in the Supplemental material (S5 and S6).

## Discussion

This study assessed the association between loss or reduction of employment and the increase of relational conflicts in a sample of Canadian adults and the extent to which this relationship was influenced by the participant’s sex and/or by COVID-19-related stress levels. To achieve this, we analysed a sample of Canadians (working full-time, part-time and/or being self-employed before the pandemic) through the iCARE study, captured from October 29, 2020, to March 23, 2021. The outcomes revealed that participants who experienced loss or reduction of employment were more likely to report an increase in relational conflicts (physical and verbal fights with people in the household). Furthermore, findings indicate that sex influences the relationship between loss or reduction of employment and relational conflict. Both males and females who experienced loss or reduction of employment were more likely to report relational conflicts compared to males and females with stable employment. However, males with loss or reduction of employment were more likely to report relational conflicts compared to females, highlighting the greater impact of these factors on males. There was a main effect of COVID-19-related stress levels on relational conflicts; however, COVID-19-related stress levels did not influence the association between the two main variables (loss or reduction of employment and relational conflicts).

The association between loss or reduction of employment and relational conflicts is consistent with the study of Langhinrichsen-Rohling et al. (2022), conducted during the COVID-19 pandemic, which showed that unemployed individuals, as well as those without access to health insurance or social support, were more likely to experience relational conflicts. These results suggest that, during a health crisis like COVID-19, income and employment stability are important to avoid relational conflict because it reduces financial strain (Buck and Neff, 2012; Conger et al., 1999; Langhinrichsen-Rohling et al., 2022; Williamson et al., 2013). Many studies indicate that financial problems are associated with dysfunctional relationships (Buck and Neff, 2012; Conger et al., 1999). The ‘stress spillover’ concept postulates that external stressors, as financial problems, predict a decrease in relationship satisfaction because individuals tend to experience more psychological distress and have fewer positive interactions with their social environment (Buck and Neff, 2012; Conger et al., 1999; Repetti, 1989). Our findings further support this notion, indicating that COVID-19-related stress was associated with increased relational conflicts among our participants. Indeed, individuals facing external stressors (e.g. public health emergency) are more likely to harbour negative attributions about their partners, employ less effective communication strategies and exhibit more maladaptive relationship behaviours, including conflicts (Neff and Karney, 2004; Randall and Bodenmann, 2009; Williamson et al., 2013). In a related context, the insecurity regarding employment status during COVID-19 has been associated with an increase in mental health disorders (Antino et al., 2022). A longitudinal study demonstrated that individuals who experienced employment insecurity during the pandemic also experienced increased anxiety and depression symptoms as well as sleep disturbances (Antino et al., 2022). Anxiety and depressive disorders, as shown in our results, have influenced the frequency of relational conflicts, aligning with literature that highlights the connection between social relationship quality and mental health (Pieh et al., 2021; Teo et al., 2013). In other words, good mental health could be associated with higher-quality relationships and, therefore, fewer conflicts (Pieh et al., 2021; Teo et al., 2013).

Males were 5.2 times more likely to report relationship conflicts compared to males without such a change, which was higher than the rate in females in the same situation. Paul and Moser’s (2009) study supports our findings and mentions that males are generally more negatively impacted by unemployment. As paid employment remains strongly linked to males’ social status in Western societies, theories of male backlash and instrumental violence suggest that in cases where males are unemployed while their partners are employed, some males may seek to reassert dominance and their traditional male role within the household, potentially resorting to violence (Eckhard, 2022; Henke and Hsu, 2022). Therefore, when males face unemployment, they are more likely to experience stigma, a decrease in self-esteem and even an increase in depressive symptoms, which can negatively impact relationship satisfaction (Álvaro et al., 2019; Eckhard, 2022; Paul and Moser, 2009). Moreover, in response to a stressor, such as job loss, males tend to exhibit more externalized behavioural problems, such as aggression, impulsivity and/or substance abuse, which are significant factors related to conflicts and the escalation of violence within households (Catalá-Miñana et al., 2017; Jewkes, 2002; van Praag et al., 2009).

The results showing a different association between loss or reduction of employment and relational conflicts by participant’s sex may be explained by the fact that males still earn a higher average income than females (Bonikowska et al., 2019; Statistics Canada, 2022a) and that married or cohabiting females may have received financial support from their husbands/partners. This financial assistance may have reduced the financial stress associated with job/income loss and, consequently, the frequency of relational conflicts. Nevertheless, females facing job loss or reduction reported more relational conflicts than those without changes and living with children was also linked to increased conflict risks. The implementation of public health measures, such as school and daycare closures, placed additional domestic, childcare and homeschooling responsibilities on families, primarily falling on females (Alon et al., 2020; Lee et al., 2021; Reichelt et al., 2021). This heightened responsibility may have contributed to greater dissatisfaction and disputes (Alon et al., 2020; Lee et al., 2021; Reichelt et al., 2021). Xue et al. (2021) further noted that females, especially single mothers, who spent extensive time on household tasks and childcare during the pandemic, reported higher levels of psychological distress, potentially exacerbating relational conflicts. However, the results indicate that loss or reduction of employment, particularly among males, was a significant risk factor associated with relational conflicts.

Finally, findings from this study show that the COVID-19-related stress level was associated with more relational conflicts, seemingly independent of job status. As previous studies showed, the pandemic contributed to elevated stress levels across various populations, with the extent of stress having a direct effect on people’s mood, well-being and behaviours, while also negatively affecting relationship functioning (Luetke et al., 2020; Shahabi et al., 2023). Indeed, the pandemic ushered in substantial changes in people’s daily lives, including reduced engagement in physical activities and hobbies, limited access to ‘non-essential’ mental health and healthcare services, separation from loved ones and being confined to home (Luetke et al., 2020). Additionally, people had to navigate new responsibilities related to childcare, home-schooling for children and work lives (e.g. working virtually from home, essential workers, etc.) (Luetke et al., 2020). These dramatic routine changes for individuals and families may have contributed to an increase in stress and the exacerbation of relational conflicts during COVID-19 (Luetke et al., 2020). Furthermore, the vulnerability-stress-adaptation model, as applied to the pandemic context by Pietromonaco and Overall (2021), illustrates how COVID-19-related stressors, coupled with pre-existing vulnerabilities such as lower socioeconomic status or attachment style, can impact various aspects of relationships, including processes, quality and stability both during and after the pandemic (Gresham et al., 2021; Pietromonaco and Overall, 2021). COVID-19-related external stressors are more likely to intensify harmful dyadic dynamics because they may create an environment in which partners find it increasingly challenging to be responsive to one another due to distractions, fatigue, or feeling overwhelmed (Pietromonaco and Overall, 2021). This disruption in partner interactions can lead to a breakdown in adaptive relationship processes, potentially resulting in more maladaptive behaviours like negativity and hostility (Gresham et al., 2021; Pietromonaco and Overall, 2021). However, the COVID-19-related stress levels did not influence the association between loss or reduction of employment and relational conflicts. One potential explanation could be that COVID-19-related stress, may not have stemmed from job loss or reduction, as the Canadian Emergency Response Benefit (CERB) provided by the Canadian government during the pandemic may have elevated any financial aspect of COVID-19-related stress. Indeed, >35.2% of workers aged 15 and over received CERB payments in 2020, averaging 17 weeks (Statistics Canada, 2021b). The percentage of CERB beneficiaries was 37.7% in Quebec, 35.8% in Ontario, 35.8% in Alberta and 34.3% in British Columbia (Statistics Canada, 2021b). Thus it is plausible that the distinctive circumstances of COVID-19 and the stress it induced through the preventative measures may have been more impactful than pandemic-induced employment changes.

## Limitations and strengths

This study has certain limitations that may influence the interpretation of the results. First, we did not use longitudinal data for the study, but consecutive cohorts drawn from the same participant pool, which limits the ability to explore temporal trends in the same individuals. Second, since all impacts were self-reported by the respondents, they may be subject to some degree of response social desirability bias. However, all iCARE surveys were completely anonymous, which reduces desirability pressures. Third, the responses only reflected those of respondents and not their partners or family members (nor their employment status), whose perspectives and experiences may differ from those of the respondents. Fourth, a distinction between individuals who were married and those who were cohabitating was not made in our analysis (the selected survey waves did not assess marital status; it was added in the latest versions of the questionnaire), potentially overlooking nuanced differences in relational dynamics. Lastly, the study assessed the extent to which the pandemic impacted participants’ relational conflicts using crude rather than validated measures, which were beyond the scope of the study. However, questions were designed to be ecologically valid and allow for easy comparisons across individuals from different countries, which is the larger context of the iCARE Study.

Despite the limitations, this study has several strengths. Firstly, it addressed relational conflicts and their implications for domestic violence. To date, this study is one of the first to have explored the impact of COVID-19 preventive measures on relational conflicts in Canada. This is also a relevant and timely topic, given the significant number of domestic violence victims in Canada with an alarming increase in reported incidents during the pandemic (Henke and Hsu, 2022; Statistics Canada, 2022, 2021a). It is therefore imperative to address the underlying causes of conflict, such as job losses and stress, to mitigate their impacts. Lastly, the large representative Canadian sample has equal proportions of males and females, which enhances the analysis by sex.

## Conclusion

In conclusion, these results show an increase in relational conflicts among individuals reporting loss or reduction of employment in both sexes, especially among males, while also underscoring COVID-19-related stress levels as a contributing risk factor for relational conflicts. The research findings highlight the importance of developing targeted prevention programmes to help families better manage the impacts associated with loss or reduction of employment, especially among males, to reduce relational conflicts and prevent the escalation of the potential of violence in intimate partner relationships. In future research, it would be valuable to establish a more precise measure of relational conflicts to delineate better the type of conflict (verbal or physical), its intensity, and even its frequency. Exploring dyads (e.g. employed males vs unemployed females) could also offer valuable insights into the intricacies of relational dynamics. Finally, it would also be relevant to examine the relationship between relational conflicts and other variables such as substance use disorders, which are associated with increased conflicts in the scientific literature.

## Supplemental Material

sj-docx-1-hpq-10.1177_13591053241260672 – Supplemental material for Relational conflicts during COVID-19: Impact of loss and reduction of employment due to prevention measures and the influence of sex and stress (in the iCARE study) Supplemental material, sj-docx-1-hpq-10.1177_13591053241260672 for Relational conflicts during COVID-19: Impact of loss and reduction of employment due to prevention measures and the influence of sex and stress (in the iCARE study) by Noémie Tremblay, Camille Leger, Frédérique Deslauriers, Lydia Hébert-Auger, Vincent Gosselin-Boucher, Simon L. Bacon, Maximilien Dialufuma Vakambi and Kim L. Lavoie; for the iCARE Study Team in Journal of Health Psychology
