# Supplementary material for: Relational conflicts during COVID-19: Impact of loss and reduction of employment due to prevention measures and the influence of sex and stress (in the iCARE study)
Source: J Health Psychol. 2024 Aug 15;30(4):749–64. doi: 10.1177/13591053241260672 (PMC11927013; doi:10.1177/13591053241260672)
Supplement: sj-docx-1-hpq-10.1177_13591053241260672 – Supplemental material for Relational conflicts during COVID-19: Impact of loss and reduction of employment due to prevention measures and the influence of sex and stress (in the iCARE study) [file sj-docx-1-hpq-10.1177_13591053241260672.docx]

**SUPPLEMENTARY MATERIAL**

***Supplementary Table S1.*** *Comparison of weighted sociodemographic characteristics from all surveys (3 to 5)*

| Weighted sociodemographic characteristics | Surveys (3 to 5) | | |
| --- | --- | --- | --- |
|  | 3 | 4 | 5 |
|  | % (n) | | |
| **Gender**  Male  Female  Missing value  **Age** | 52,3 (886)  47,7 (808)  1 | 51,1 (872)  48,9 (834)  5 | 50,4 (853)  49,6 (840)  4  33,7 (567)  57,2 (962)  9 (152)  15  66,4 (1115)  33,6 (564)  17 |
| 51 years old and more  26-50 years old  Less than 25 years old  Missing value  **Education Level**  High school diploma or less  College or more  Missing value | 32,8 (552)  56,7 (953)  10,4 (175)  16  65,3 (1089)  34,7 (579)  28 | 35 (588)  54,8 (921)  10,2 (170)  32  65,9 (1106)  34,1 (573)  32 |  |
| **Region (Province)** |  |  |  |
| Ontario | 35,8 (608) | 39,1 (668) | 37,2 (631) |
| Québec | 24,7 (418) | 25,4 (435) | 23,8 (403) |
| British Columbia | 14,8 (250) | 12,9 (221) | 13,3 (225) |
| Alberta | 12,3 (208) | 10,5 (179) | 12,1 (206) |
| Saskatchewan | 3,4 (57) | 2,9 (50) | 3,2 (54) |
| Manitoba | 2,8 (47) | 2,4 (41) | 3,6 (61) |
| Nova Scotia | 2,3 (38) | 3,6 (61) | 2,9 (49) |
| New Brunswick | 2,4 (41) | 2,3 (39) | 2 (34) |
| Newfoundland | 1,5 (25) | 0,8 (13) | 1,6 (27) |
| Prince Edward Island | 0,2 (3) | 0,2 (4) | 0,4 (7) |
| **Region Type** |  |  |  |
| Suburban or suburb | 46,3 (16) | 44 (14) | 18,3 (6) |
| Urban or city  Rural or countryside | 39,1 (13)  14,6 (5) | 20,2 (6)  35,8 (11) | 47,9 (16)  33,8 (11) |
| Missing values | 1662 | 1680 | 1664 |
| **Income** |  |  |  |
| 60K/year or more | 61 (929) | 59,8 (933) | 60,8 (938) |
| Less than 60K/year  Missing values | 39 (595)  172 | 40,2 (627)  150 | 39,2 (604)  155 |
| **How many children (under 18 years old) live with you at home** | | | |
| No children | 66 (1103) | 66,3 (1111) | 66,9 (1097) |
| At least one child | 34,1 (569) | 33,7 (566) | 33,1 (544) |
| Missing values  **Current employment status** | 24 | 34 | 56 |
| Employed | 90,2 (1474) | 86,1 (1434) | 88,1 (1468) |
| Unemployed | 9,8 (161) | 13,9 (231) | 11,9 (198) |
| Missing values | 61 | 46 | 31 |
| **Main Pre-pandemic Employment Sector** |  |  |  |
| Professional | 22,7 (321) | 23,5 (323) | 24,5 (390) |
| Service and sales worker | 17,4 (246) | 17,8 (244) | 16,5 (262) |
| Manager | 13,4 (191) | 14,4 (197) | 13 (207) |
| Technician or associate professional | 9 (127) | 9,8 (135) | 7,6 (121) |
| Craft and related trades | 5,7 (81) | 4,3 (59) | 4,8 (77) |
| Plant, machine operator and assembler | 5,2 (73) | 4,2 (58) | 5,1 (81) |
| Elementary occupations | 3,5 (50) | 4,3 (59) | 5,5 (87) |
| Agriculture, forestry, and fishing worker | 2 (28) | 2,1 (29) | 1,8 (29) |
| Public service worker | 0,8 (12) | 1,5 (21) | 0,7 (12) |
| Armed forces occupations | 0,7 (10) | 1,2 (17) | 0,4 (7) |
| Other | 0,1 (1) | 0,9 (12) | 3,1 (49) |
| Missing values | 277 | 339 | 105 |
| **Presence of any depressive disorders** |  |  |  |
| No | 83,3 (1367) | 83,6 (1373) | 85 (1412) |
| Yes | 16,8 (275) | 16,5 (270) | 15 (249) |
| Missing values | 54 | 68 | 36 |
| **Presence of any anxiety disorder** |  |  |  |
| No | 79 (1292) | 75,2 (1237) | 80,3 (1337) |
| Yes | 21 (344) | 24,8 (407) | 19,7 (329) |
| Missing values | 60 | 67 | 31 |
| **COVID-19-related stress level** |  |  |  |
| Increase | 73 (1219) | 71,1 (1190) | 66,2 (1094) |
| No increase | 27 (452) | 28,9 (484) | 33,8 (559) |
| Missing values | 25 | 37 | 44 |

| Variables | **Loss/reduction of employment** | **Sex** | **COVID-19-related stress levels** | **Age** | **Survey wave** | **Ethnicity** | **Any depressive disorder** | **Any anxiety disorder** |
| --- | --- | --- | --- | --- | --- | --- | --- | --- |
| **Loss/reduction of employment** | 1.00 | 0.03 | 0.27 | -0.09 | -0.02 | -0.12 | -0.11 | -0.14 |
| **Sex** |  | 1.00 | 0.16 | -0.12 | 0.02 | 0.04 | -0.08 | -0.15 |
| **COVID-19-related stress levels** |  |  | 1.00 | -0.17 | -0.05 | -0.07 | -0.17 | -0.22 |
| **Age** |  |  |  | 1.00 | -0.01 | 0.17 | 0.08 | 0.11 |
| **Survey wave** |  |  |  |  | 1.00 | 0.03 | -0.01 | -0.01 |
| **Ethnicity** |  |  |  |  |  | 1.00 | -0.05 | -0.04 |
| **Any depressive disorder** |  |  |  |  |  |  | 1.00 | 0.55 |
| **Any anxiety disorder** |  |  |  |  |  |  |  | 1.00 |

***Supplementary Table S2.*** *Spearman Correlation Coefficients (values close to +1 indicate a strong positive relationship, values close to -1 indicate a strong negative relationship and values close to 0 indicate little to no relationship)*

| **Variables** | **Variance Inflation Factors (VIF)** |
| --- | --- |
| Loss or reduction of employment | 1.13 |
| COVID-19-related stress levels | 1.20 |
| Age | 1.07 |
| Sex | 1.05 |
| Survey wave | 1.00 |
| Ethnicity | 1.05 |
| Any depressive disorder | 1.51 |
| Any anxiety disorder | 1.56 |

***Supplementary Table S3.*** *Multicollinearity analysis: Variance Inflation Factors*

***Supplementary Table S4.*** *Association between loss or reduction of employment and relational conflicts (and covariates) influenced by sex**

| Variables | DF | Wald Chi-Square | p-value |
| --- | --- | --- | --- |
| **Loss or reduction of employment**  (reference: no loss or reduction of employment) | 1 | 113.33 | **<.001** |
| **Sex**  (reference: males) | 1 | 0.94 | 0.331 |
| **Loss/reduction of employment*sex** | 1 | 10.16 | **<.005** |
| **Age**  (reference: 51 years old and more) | 2 | 23.43 | **<.001** |
| **Ethnicity**  (reference: white) | 1 | 18.60 | **<.001** |
| **COVID-19-related stress levels**  (reference: no stress) | 1 | 152.33 | **<.001** |
| **Wave number** (reference: wave 5) | 2 | 0.18 | 0.915 |
| **Depressive disorder**  (reference: no depressive disorder) | 1 | 10.66 | **<.005** |
| **Anxiety disorder**  (reference: no anxiety disorder) | 1 | 1.84 | 0.175 |
| **Living with children (under 18 years old)**  (reference: living without children) | 1 | 10.34 | **<.005** |

OR: Odds Ratio, CI: Confidence interval, DF: Degree of freedom

**Adjusted for age, survey wave, ethnicity (white vs. other), the presence of anxiety/depressive disorders, and the weighted variable*

***Supplementary Table S5.*** *Association between COVID-19-related stress levels and relational conflicts (and covariates)**

| Variables | OR (95%CI) | DF | Wald Chi-Square | p-value |
| --- | --- | --- | --- | --- |
| **Loss or reduction of employment**  (reference: no loss or reduction of employment) | 2.63 (2.20-3.14) | 1 | 113.88 | **<.001** |
| **COVID-19-related stress levels**  (reference: no stress) | 9.54 (6.70-13.60) | 1 | 155.87 | **<.001** |
| **Sex**  (reference: males) | 0.87 (0.73-1.03) | 1 | 2.61 | 0.106 |
| **Age**  (reference: 51 years old and more) |  | 2 | 22.68 | **<.001** |
| 26-50 years old | 1.55 (1.25-1.93) |  |  | **<.001** |
| Less than 25 years old | 1.93 (1.43-2.60) |  |  | **<.001** |
| **Ethnicity**  (reference: white) | 1.55 (1.27-1.89) | 1 | 18.67 | **<.001** |
| **Wave number** (reference: wave 5) |  | 2 | 0.14 | 0.934 |
| 3 | 0.98 (0.79-1.21) |  |  | 0.873 |
| 4 | 1.02 (0.83-1.26) |  |  | 0.836 |
| **Depressive disorder**  (reference: no depressive disorder) | 1.51 (1.17-1.94) | 1 | 10.34 | **<.005** |
| **Anxiety disorder**  (reference: no anxiety disorder) | 1.17 (0.93-1.48) | 1 | 1.81 | 0.178 |
| **Living with children (under 18 years old)**  (reference: living without children) | 1.35 (1.12-1.62) | 1 | 10.37 | **<.005** |

OR: Odds Ratio, CI: Confidence interval, DF: Degree of freedom

**Adjusted for age, survey wave, ethnicity (white vs. other), the presence of anxiety/depressive disorders, and the weighted variable*

***Supplementary Table S6.*** *Association between loss or reduction of employment and relational conflicts (and covariates) influenced by COVID-19-related stress levels*

| Variables | DF | Wald Chi-Square | p-value |
| --- | --- | --- | --- |
| **Loss or reduction of employment**  (reference: no loss or reduction of employment) | 1 | 20.08 | **<.001** |
| **COVID-19-related stress levels**  (reference: no stress) | 1 | 142.30 | **<.001** |
| **Loss/reduction of employment*stress** | 1 | 0.46 | 0.497 |
| **Age**  (reference: 51 years old and more) | 2 | 22.59 | **<.001** |
| **Ethnicity**  (reference: white) | 1 | 18.74 | **<.001** |
| **Sex**  (reference: male) | 1 | 2.59 | **0.11** |
| **Wave number** (reference: wave 5) | 2 | 0.13 | 0.936 |
| **Depressive disorder**  (reference: no depressive disorder) | 1 | 10.32 | **<.005** |
| **Anxiety disorder**  (reference: no anxiety disorder) | 1 | 1.77 | 0.183 |
| **Living with children (under 18 years old)**  (reference: living without children) | 1 | 10.37 | **<.005** |

OR: Odds Ratio, CI: Confidence interval, DF: Degree of freedom

**Adjusted for age, survey wave, ethnicity (white vs. other), the presence of anxiety/depressive disorders, living with children (under 18 years old) and the weighted variable*
